# Resolution on Interchange of License

**Published:** 1903-10

**Authors:** 


### Domestic Correspondence.

### RESOLUTION ON INTERCHANGE OF LICENSE.

To the Editor:

Sir,—The following is a resolution passed by the Board of Examiners of New Jersey and also passed unanimously by the National Board at their meeting at Asheville. Two States have already entered into an agreement on this plan,—viz., Ohio and Indiana.

C. S. Stockton.

Newark, N. J., August 5, 1903.

"*Resolved,* That an interchange of license to practise dentistry be and is hereby recommended to be granted by the various State boards on the following specific conditions:

"Any dentist who has been in legal practice for five years or more and is a reputable dentist of good moral character, and who is desirous of making a change of residence into another State, may apply to the examining board of the State in which he resides for a new certificate, which shall attest to his moral character and professional attainments, and said certificate, if granted, shall be deposited with the examining board of the State in which he proposes to reside, and the said board, in exchange therefor, may grant him a license allowing him to practise dentistry."

